# Adolescent Nutrition and Health: a Critical Period for Nutritional Intervention to Prevent Long Term Health Consequences

**DOI:** 10.1007/s13668-025-00706-4

**Published:** 2025-10-23

**Authors:** Janaki Parajuli, Pattaneeya Prangthip

**Affiliations:** Department of Tropical Nutrition and Food Science, Faculty of Tropical Medicine, Mahidol University, Bangkok, Thailand

**Keywords:** Adolescence, Nutrition, Malnutrition, Obesity, Cardiometabolic risk, Dietary patterns, Interventions, Public health

## Abstract

**Purpose of Review:**

This review aims to examine adolescent nutrition as a critical and often neglected component of global health agendas. Adolescence represents a period of rapid growth and development with increased nutritional and energy demands, remaining a ‘hidden crisis’ in nutritional health. The review explores the ‘triple burden’ of malnutrition affecting this population—undernutrition, micronutrient deficiencies, and overnutrition—and analyzes the individual, social, environmental, and socio-economic factors influencing adolescent nutritional status.

**Recent Findings:**

Poor nutrition during adolescence has been linked to both immediate and long-term health consequences, including micronutrient deficiencies, obesity, and cardiometabolic conditions. Evidence suggests that adolescents’ heightened receptivity to societal influences can be leveraged through targeted interventions. Family-led programs, environmental reforms, and comprehensive policy measures have shown promising results in promoting healthier nutritional behaviors among adolescents.

**Summary:**

The nutritional challenges facing adolescents require urgent attention through comprehensive, multi-sectoral strategies. Effective interventions during this critical developmental window can foster long-term health outcomes and help break intergenerational cycles of malnutrition. This review emphasizes the importance of prioritizing adolescent nutrition within global health initiatives to address this significant and often overlooked public health concern.

## Introduction

Adolescence is a critical developmental stage, accounting for 16% of the global population with approximately 1.3 billion adolescents worldwide [1]. It is marked by significant physical, cognitive, and emotional growth, second only to infancy in developmental rate [2]. During this stage, the brain undergoes rapid development, which affects emotional regulation, information processing, and decision-making [3]. Adolescents begin to form peer-based relationships and engage in experimentation, exploration, and risk-taking behaviors [3, 4]. This period also represents a time of rapid physical development and increased nutritional demands, including higher requirements for energy, protein, iron, calcium, and other key mocronutrients, many of which exceed adult recommendations [5–7].

Despite the importance of this age group, adolescent health and nutrition remain notably neglected in global health and nutritional policies [8]. The Commission on Adolescent Health and Wellbeing has brought this critical issue to the forefront, labelling adolescent nutrition as the ‘hidden crisis’ and emphasizing the urgent need for investment in young people’s health [9]. This age group often falls into a policy gap - too old to receive the protective care given to children yet frequently overlooked in adult health initiatives. The widespread exclusion of adolescents from international policies is particularly concerning given that adolescence is generally viewed as a period of improved health and reduced vulnerability to illness, leading to its neglect in public health discussions [9]. This oversight is especially problematic as poor dietary patterns established during adolescence often persist into adulthood, contributing significantly to the global burden of non-communicable diseases (NCDs).

Adolescents’ physical, cognitive, and economic development are fundamentally shaped by their nutritional status [8, 10, 11]. Inadequate nourishment-emcompassing both undernutrition (including stunting, wasting, and micronutrient deficiencies) and overnutrition(excess caloric intake leading to overweight and obesity)- remains prevalent globally, though its specific manifestations vary by region where undernutrition remaining more common in low- and middle-income countries and overnutrition increasingly prevalent in urban and high-income settings-both forms represent significant public health concerns during adolescence [12]. The magnitude of this problem is illustrated by the global burden of iron deficiency anemia, which results in the loss of 1,161 and 1,365 disability-adjusted life years (DALYs) per 100,000 females and males aged 10–14, respectively [13]. The transition into adulthood brings additional challenges as adolescents experience dietary and lifestyle changes that increase their susceptibility to eating disorders, overweight, and obesity [14]. These nutritional challenges have far-reaching implications for population health, emphasizing the critical importance of addressing and protecting adolescents’ nutritional needs for both current and future health outcomes [15].

The nutritional landscape for adolescents is further complicated by the global double burden of malnutrition - the coexistence of undernutrition and overnutrition within the same population, household, or individual- which particularly affecting those in urban settings of low- and middle-income countries. This review provides a comprehensive examination of the global nutritional challenges faced by adolescents, emphasizing the urgency of addressing malnutrition to mitigate both immediate and long-term health risks. By analysing evidence-based strategies for improving adolescent nutrition, this review highlights the necessity of implementing comprehensive, multi-sectoral approaches to achieve sustainable public health outcomes. Understanding and addressing these nutritional challenges during this critical period is essential for breaking the intergenerational cycle of malnutrition and fostering healthier future generations.

## Methods

### Literature Search and Selection Strategy

This narrative review was conducted through a comprehensive search of peer-reviewed literature published between 2000 and 2024. The following electronic databases were searched: PubMed/MEDLINE, Web of Science, and Google Scholar. Additionally, relevant reports from international organizations (WHO, UNICEF, FAO) were included.

### Search Terms

Primary search terms included: “adolescent nutrition,” “teenage nutrition,” “adolescent diet,” “adolescent health,” combined with secondary terms such as “malnutrition,” “obesity,” “dietary patterns,” “nutritional intervention,” and “food environment.”

### Inclusion Criteria

- Original research articles, systematic reviews, and meta-analyses.
- Publications in English.
- Studies focusing on adolescents (10–19 years).
- Articles addressing nutrition, dietary patterns, or related health outcomes.
- Global and regional studies.

### Exclusion Criteria

- Case reports.
- Studies focusing solely on specific disease conditions.
- Publications without full text availability.

The selected articles were critically appraised for relevance, methodology, and significance of findings. Priority was given to large-scale studies, systematic reviews, and publications from recognized institutions. The final selection included approximately 110 references that provided comprehensive coverage of the topic while maintaining focus on the most relevant and impactful research in the field.

## Results and Discussion

### Dietary and Nutritional Characteristics of Adolescents: Adolescents Prone to Nutritional Vulnerability

Adequate nutrient intake is essential in all stages of life but particularly during the adolescent years, not only to facilitate rapid physiological growth and development but also to lay the foundations of good health for later life [16]. The demand of puberty growth necessitates higher dietary requirements during adolescence. Numerous factors, including brain development and the understanding of health-related issues, as well as the broader familial, societal, and economic milieu in which they live, eat, study, work and play, influence adolescents’ eating pattern and behaviors [17–21]. Furthermore, sixteen million girls between the ages of 17 and 19 give birth to children each year, meaning that they too need to be in a healthy enough nutritional state [22].

Adolescents are vulnerable of ‘triple burden’ of malnutrition encompassing undernutrition, micronutrient deficiency and over nutrition [23]. Undernutrition refers to the outcome of insufficient food intake and repeated infectious diseases. It includes being underweight, stunting(low height-for-age), wasting (low weight-for-height), and micronutrient deficiencies [24]. Overnutrition refers to excessive intake of energy and nutrients, leading to overweight and obesity. It is typically associated with the consumption of energy-dense, nutrient-poor foods and insufficient physical activity [24]. A study done in 200 countries among over 31 million children and adolescents between 1975 and 2016 revealed an increase in the mean BMI and incidence of overweight and obesity worldwide while the percentage of underweight children and adolescents surpasses that of those classified as overweight or obese. The burden of underweight is shifting towards South Asia and central eastern and western Africa, even while BMI is at high levels in many high-income nations and is increasing in other regions of Asia [6, 25].

Adolescents undergo rapid developmental changes that directly influence eating behaviours and nutritional vulnerability. The combination of increased autonomy in food choices, heightened peer influence, and ongoing brain development in areas responsible for decision-making creates a period of elevated risk for dietary inadequacy. These developmental factors, coupled with increased nutritional demands during growth spurts, make adolescents particularly susceptible to poor dietary patterns that may persist into adulthood [15, 26–32]. Double burden of malnutrition is more common in urban settings, disproportionately affecting women. For instance in Nairobi’s urban slums, both thinness and excess weight were observed within the same adolescent population, particularly among girls following energy-dense Westernized diet [33]. Similarly, Ethiopian female adolescents displayed 15% underweight and 8.4 overweight/obesity prevalence [34]. A study done in 200 countries among over 31 million children and adolescents between 1975 and 2016 revealed an increase in the mean BMI and incidence of overweight and obesity worldwide while the percentage of underweight children and adolescents still exceeds the percentage of overweight or obese children. The burden of underweight is shifting towards South Asia and central eastern and western Africa, even while BMI is at high levels in many high-income nations and is increasing in other regions of Asia [6, 25].

Adolescence is a second window of opportunity for development and growth during the intergenerational lifecycle [32]. Puberty typically starts for boys between 9 and 15 years of age and for girls between 8 and 14 years of age. Although there are variations worldwide, the median age at menarche is typically between 12 and 13 years old, or 2 to 2.5 years after breast growth begins [35, 36]. Girls who have higher body mass index (BMI) or taller as kid are more likely to menarche and go through puberty earlier in adulthood, especially if they already have overweight or obese as well as experience subnormal growth spurts in their adolescent height [32, 37–40]. Similarly, girls who suffer childhood stunting usually start menarche later in life and reach puberty later. These girls might, however, make up for lost development during adolescence [32, 37, 41, 42].

Girls’ growth rate frequently increases between the ages of 10 and 12, 1.5–2.5 years before to menarche. Boys usually exhibit the earliest symptoms of puberty between the ages of 10 and 16. Between the ages of 12 and 15, this is when the adolescent naturally grow the fastest. Typically, boys go through a growth spurt two years later than girls [29, 43]. The frequency, duration, and intensity of pubertal growth spurts varied which are marked by a sudden increase in height and weight, even though every teenager goes through a similar growth pattern during puberty [44–46]. Adolescents’ nutrient needs steadily rise and are more strongly correlated with growth rates and patterns (commonly referred to as the Tanner phases) than with their chronological age [45, 46]. Stunting, delayed menarche and sexual development, and decreased linear growth may occur if there is malnutrition among adolescents [28, 30, 31, 43].

Adolescence requirement for macro-and micro-nutrients increases. During the physical growth peak, there is an increased need of micronutrients like iron, iodine, calcium, vitamin A, vitamin D [30, 43, 47, 48]. An adolescent girl who is moderately active requires 2,300 kcal per day [43]. Globally, nearly one-third of women become pregnant during their adolescent years, highlighting the critical need to ensure nutritional adequacy for the health of vulnerable young women and infants. Suboptimal micronutrient status is a key nutritional challenge amongst adolescent mothers, with iron, iodine and folic acid emphasised as nutrients of particular concern [49]. Iodine deficiency among women of childbearing age may lead to intellectual impairments and cognitive and behavioural problems in offspring [50]. In South Asia, 11% of adolescent girls aged 15–19 years are too short, 39% are underweight and 55% are anaemic. The diet of the adolescent girls and women in South Asia are often too poor to meet nutritional needs for healthy growth and development. Only 20 to 40% of South Asian adolescent girls meet their recommended dietary intake [51].

Adolescents are exposed to stimulating, exciting, and stressful environments due to natural brain development, which may result in impulsive actions that can adversely affect nutritional status, including irregular meal patterns and uncontrolled eating [52, 53]. While some risk taking is developmentally appropriate, research demonstrates that specific impulsivity facets, particularly negative urgency, significantly predict poor diet quality and problematic eating behaviors under peer influence [52, 53].

## Dietary Eating Habits: Decline in Diet Quality during Adolescence

Poor diet consumption is regarded as a major modifiable risk factor for chronic diseases and is one of the main causes of the worldwide burden of non-communicable diseases [54]. Dietary and health imbalances that track across time are expected to have an impact on the routes by which poor food quality during adolescence affects health throughout life [55]. However, adolescents have been consistently identified as displaying the poorest quality diets of all populations groups, with US adolescents (ages 12–19) demonstrating the poorest adherence to Dietary Guidelines, scoring approximately 44/100 on the HEI-2010 versus 50–52 in younger children-indicating the largest gap between actual intake and recommendations among all age groups [56, 57]. Moreover, recent studies focusing on adolescents in low-and middle-income countries have highlighted insufficient dietary intake from essential food sources such as meat, fruits, vegetables, milk products, and protein while there is increasing trend towards the consumption of nutrient-poor diets, including fast food, excessive snacking, and sugary treats [58–61]. A recent review study shows that Asian pre-school children adhering to the westernized eating pattern, which is described as a high intake of sugary beverages, snacks, and red meat was found to have significantly higher odds of being obese [62].

Adolescents’ diet quality has improved somewhat in recent years: US youths’ diet quality scores from 1999 to 2016 showed a modest improvement, with a decrease in added sugar and sugar-sweetened beverages and an increase in wholegrain, fruit, vegetables, poultry and eggs. However adolescents continue to struggle with low diet quality, with the highest prevalence of poor diet quality in USA among those aged 12–19 [63]. According to an analysis of trends in US teenage dietary intakes, ultra-processed food consumption has increased recently; in 2018, ultra-processed foods accounted for 68% of adolescent energy intake. Although, food’s degree of processing is not a predictor of its healthfulness, nutrient profiling of the meals ingested did indicate that foods classified as ultraprocessed compared to unprocessed foods had lower levels of protein and fiber and a higher proportion of carbohydrates, added sugars, and sodium [64]. Intake of highly processed foods is similarly pervasive among South Asian adolescents, with high intake of chips, snacks, white bread, soft drinks, etc. In one study conducted in Nepal, showed only small proportion of adolescents (4%) adequately consumed fruits and vegetables while an overwhelming proportion (42%) consumed processed and junk food at least twice a week [65]. Most of the adolescents during their mealtime eat junk food and get addicted to its taste, but it has low nutritive value and high calories, which results in obese children. Junk foods are also laced with colours which are often edible, carcinogenic and harmful to the body which can affect the digestive system. Food colouring can cause hyperactivity and lapses of concentration in children, resulting in a child with learning disabilities [66]. The report, provided by the Office of Environmental Health Hazard Assessment (OEHHA) under the California Environmental Protection Agency evaluated the neurobehavioral effects of synthetic food dyes in children which concluded that synthetic food dyes can impact neurobehavior in some children and calls for updated regulatory standards and further research to protect vulnerable populations [67]. Although these findings raise safety concerns, evidence in adolescents, remains limited and inconclusive, warranting further investigation.

Adolescence is a pivotal time of personal development, in which lifelong traits and habits can be discovered and established. This life stage has been described as a “window of opportunity” for the development of dietary patterns, and this stage of life has been emphasized as crucial but underappreciated period for the construction of long-lasting health behaviours [68]. Adolescent dietary behaviours have displayed the strongest continuation into adulthood [69]. Adolescents who adhere the most closely to dietary patters, such as ‘’traditional’’, health-conscious’’, ‘’high protein’’, ‘’high fat’’ and ‘’vegetarian-style”, show a notable ability to track similar dietary pattern 20 years later, according to some longitudinal cohort studies [70, 71]. Still, establishment of healthful dietary patterns is imperative in meeting the nutritional requirements of adolescence and could potentially aid in the creation of lifelong healthy behaviours.

## Potential Risk Related To Adolescent Nutrition and Health: Causes and Consequences

### Adequate Nutrient Intake is Essential in All Stages

**Fig. 1:**
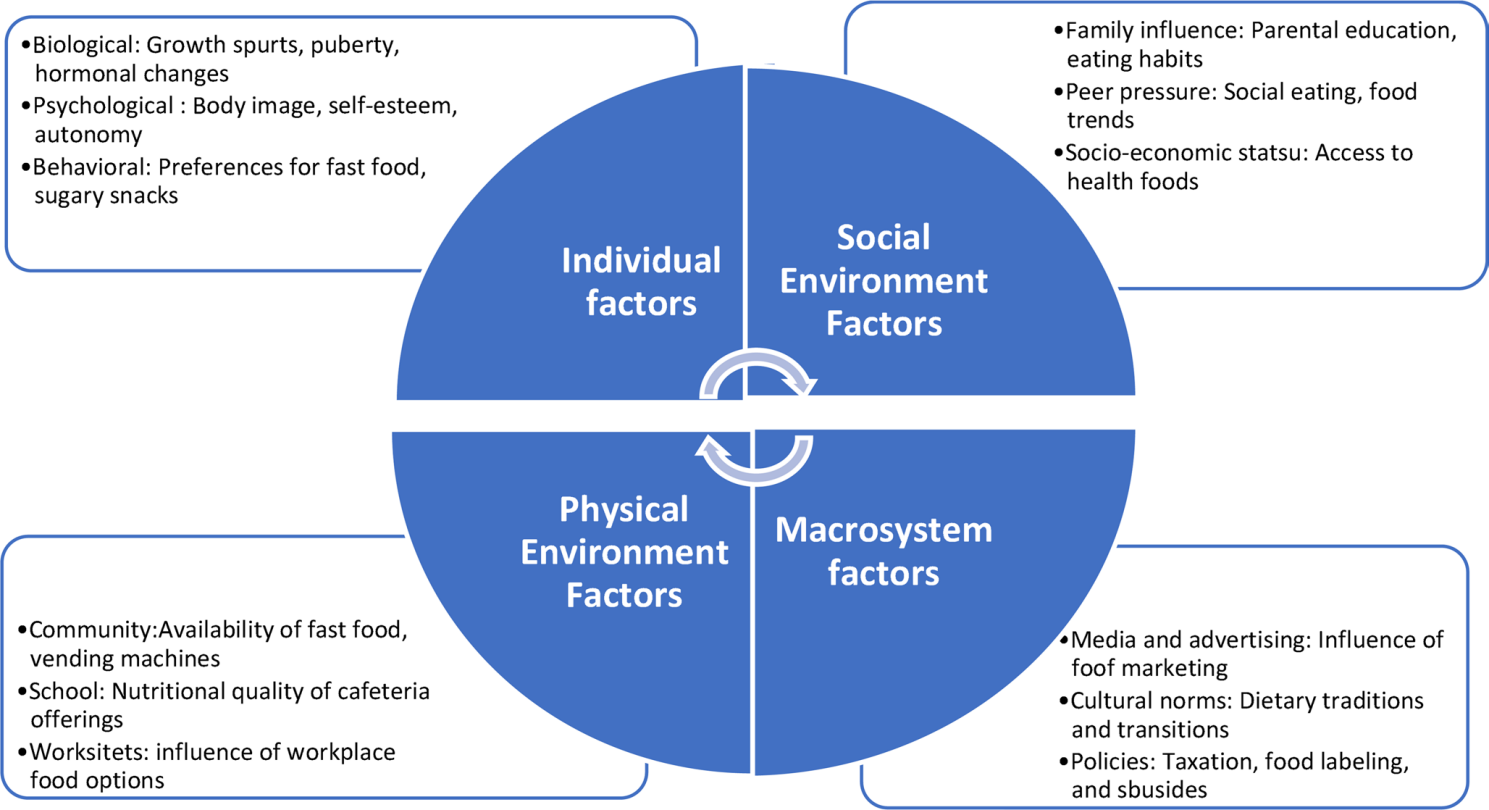
Graphical Chart showing key factors affecting adolescent nutrition

Nutrition being the fundamental component of health and well-being, particularly during adolescence, many adolescents struggle with poor dietary habits, due to a complex interplay of individual, social, environmental, physical and microsystem influences [71]. Key factors affecting adolescent nutrition are in Fig. 1.

#### Individual (Intrapersonal) Factors Influencing Adolescent Nutrition and Health

Adolescence is one of the most dynamic and complex transitions in the lifespan. It has been considered as a period of ‘storm and stress’ in which disruptions in mood, increases in risk-taking behaviour, and increased conflict with parents/guardians can contribute to turbulent thoughts, emotions, and behaviours [72]. This age group brings drastic changes in lifestyle, eating behaviours, and increased exposure to environmental influences, all of which can increase the nutritional vulnerability [73]. Personal factors like body image, preferences, degree of autonomy, interest in health, and nutrition knowledge have been identified as important considerations for health and food choices in adolescents [74]. Moreover, there are numerous psychosocial factors including attitudes, beliefs, self-efficacy, food preferences, taste and sensory perception of food, priority of health and nutrition, knowledge regarding food, mood and mental health considered as the key indicators of food choices among individuals (Robson et al., 2000). Similarly, biological determinants such as sex, hunger, taste, appetite, weight status and allergies strongly influence the diet pattern of the adolescence [75]. Intention and the sedentary behaviour also are the strongest determinants affecting the dietary pattern of the adolescents [76].

#### Social Environmental (Intrapersonal) Factors Influencing the Adolescent Health and Nutrition

Adolescents’ eating behaviors are strongly influenced by their social environments, which include family, friends, and peer networks. Interpersonal processes and relationships within the family and with friends, neighbours, and acquaintances all have a substantial impact on food choices and eating behaviours [71]. Adolescence is a time when the “social brain” develops, making this age group especially sensitive to and aware of how other people affect their decision-making [77]. The family is the major influence in the adult eating behaviours.The family is a provider of food, and the family influences food attitudes, preferences, and values that affect lifetime eating habits.As they transition towards greater independence and autonomy, food habits of adolesccents reflect the changing role of parental influence on food choices [78]. Parental characteristics such as education level, social class, income, health, marital relationships, parenting style, stress, efficacy, habits and preferences have been identified as the important factors influending on the lives, and indeed the diets, of adolescents [79]. Children who have obese parents are at increased risk for obesity. This increasded risk is partly genetic and is due to parental modeling of healthy behaviors and characteristics of the home environment (for example, access to healthy food and opportunities for physical activity [80].

Adolescent behaviour is greatly influenced by their peers. They contribute to the establishment of behavioural norms, especially those pertaining to peer group acceptance. Adolesents spend a lot of time with their friends, and eating is an important form of socialization and recreation. Peer pressure and group conformity are thought to be significant factors in determining food acceptablility and selection since teenagers strive for social identity and peer approval [71].

Social class and socioeconomic status are amongst the strongest determinants of healht-related behaviours. Poverty is found to be strongly associated with unhealthy weight status and dietary intake. Adolescents living in poverty represent an important target group for further health interventions aimed at improving diet and weight status. The exposure of poor adolescents to a diet high in energy and low in nutrients is a concern and may have implications for their long-term health [81]. A strong income gradient of weight status and dietary intake has been repeatedly observed, with those who experience greater levels of deprivation consistently displaying poorer quality diets and the highest risk of overweight and obesity [82]. Thus, adolescents’ interactions with their surroundings are influenced by their socioeconomic status and financial resources, those who are socially disadvantaged are more susceptible to obesogenic situation due to the lack of choice and less financial freedom [83].

#### Physical Environmental Influences (Community Settings) Influencing Adolescent Nutrition and Health

The physical environment within the community influences accessibility and availability of foods. Community settings most proximal to adolescents and influential in affecting their food choices includes schools, fast-food outlets, restaurants, shopping malls, vending machines and convenience stores [71]. Adolescents consume significantly more “non-core”, hight-sugar, high-fat foods outside of their homes [84]. The school and the retail environments are frequently overstocked with inexpensive, enticing, nutrient-poor hight-energy foods [85]. The food environment in schools and their immediate vicinities associated with excess weight in adolescents. While these findings arecorrelational, promoting healthy food programs and improving the availability of nutritious options within school settings may represent a promising approach for supporting healthier dietary behaviours, which in turn could help mitigate the risk of excess weight [86].

The growing trend of commercialism and aggressive marketing in schools like direct advertising in schools, school bus advertising for soft drinks and fast-food establishments, ‘’free’’ testbook covers advertising candy, chips and soft drinks, and product give away in coupons and samples. Such environment provide easy asccess to high-fat and high-sugar food and beverages products in school and in direct conflict with the goals of nutrition education [87]. Similarly the presence of vending machine in school is related to some weight status, diet and meal behaviours among adolescents [88]. Apart from these, convenience stores and fast-food outlets are oftern located near school buildings and recreaton centers, making them convenient and accessible unhealthy food sources. Worksites will also have an impact on the food choices and dietary quality of the adolescents because many fast-food restaurants and convenience stores employ adolescents. These worksites will influence their food intake as many employees receive food discounts, free beverages, and eat meal on site during work shifts (Robson et al., 2000).

#### Macrosystem Influences Influencing Adolescent Health and Nutrition

Macrosystem factors play a more distal and indirect role in determining food behaviours. It includes mass media and advertising, social and cultural norms around eating, food production and distribution systems, which influence food availability; and local, state and federal policies and laws that regulate or support food-related issures, such as availability and pricing. Online and social media are increasingly becoming the primary avenues for adolescent exposure to food messaging and marketing. From peer posting of food images, prolific food advertising, product placement, and increasing use of online food delivery services, the exposure of adolescents to readily available unhealthy food products is just as, if not more, pervasive in the online space as in the physical environment [89]. Adolescent health must be protected by taking into account and addressing every aspect of the contemporary eating enviornment.

### Consequences of Poor Nutrition and Dietary Intake in Adolescence

#### Overweight/Obesity

Adolescence is a critical stage in the development of obesity and for starting risk factors for other chronic metabolic diseases in adulthood [90]. Recent studies in 35 countries and regions in the WHO European Region and North America on health behaviours among school-aged children showed that the food habits of adolescents are characterised by high consumption of sweets and soft drinks, skipping breakfast and low consumption of fruits and vegetables [91] which contribute to rapid increase in obesity and other risk factors for non-communicable diseases. The issue of adolescent overweight/obesity is not confined only to high –income countries, with an estimated 21.4% in low to middle-income countries affected by overweight/obesity [92].

Overweight/Obesity in adolescence has long-term associations with adult weight status, with consistently linked to a higher risk of oberweight and obesity in adulthood, according to the longitudinal research [93]. Obesity in adolescence also has immediate physical effects, including musculoskeletal issues, obstructive sleep apnea, and psychological impacts [94]. Moreover, obesity is associated with substantial mental health burden and decreased quality of life, with adolescents living with obesity displaying an increased presentation of psychological comorbidies including depression, anziety and low self-esteem [95].

Screen exposure influences risk of obesity in children and adolescents via increased exposure to food marketing, increased mindless eating whild watching screens, displacement of time spent in more physical activities, reinforcement of sedentary behaviours, and reduced sleep time [96]. Additionally, from a psychosocial perspective, it is considered as a period of increased risk of depression and anxiety [97].

It is suggested that prevention of obesity in adolescents should be the public health priority. As adolescent start interacting with their surroundings, they are exposed to more risk factors for obesity during this period. Given how quickly the prevalnece of overweight and obesity among adolescents has increased, it is more likely that present trends are due to significant changes in the modern food environment and lifestyles than to biological changes [98]. As the prevalence of adolescents affected by overweight is currently greater than the prevalence of those living with obesity, there is vast potential for early obesity preventioni startegies targeting youth with overweight who are at high risk of developing obesity [99]. Obesity is a multifactorial disease, and various genetic, behavioral and sociocultural characteristics can affect its development; thus, early management of overweight and obesity by altering eating behaviour and physical activity may be critical. Therefore, a drastic scaling up of obesity treatment and prevention is required to subside and prevent the epidemic of adolescent obesity.

#### Development of Metabolic Risk Factors

Poor diet quality and overweight/obesity are identified as important risk factors for the development of various preventable non-communicable diseases (NCDs) later in adulthood [100]. Many studies have shown links between eating a poor-quality diet and elevated metabolic and cardiovascular risk biomarkers in adolescents [101–103]. However, other analyses suggest that cardiometabolic risk factors only manifest in late adolescence or later in life, suggesting that the effects of a poor diet on metabolic health may not always be evident right away [104].

A prospective study tracking five cardiovascular risk factors from childhood to adulthood showed associations between early-life exposures and adult cardiovascular events by age 40 [105]. Despite long-standing screening guidelines [106], lipid assessments in children are still infrequent [107]. This highlights the need for early detection and interventions to support the primordial prevention of metabolic diseases.

#### Impaired Growth and Development in Adolescents Due To Deficiencies in Nutrition

Adequate intake of energy and nutrients is essential for adolescent growth. Micronutrients support metabolic functions (e.g., B vitamins), bone mineralization (calcium), hemoglobin production(iron), and cellular growth(zinc). Deficiencies in vitamin A, iron, and iodine remain prevalent in developing countries, impairing adolescent health and educational performance, and hindering economic growth [108]. Vitamin D deficiency, affecting upto 80% of Europeon adolescents, is associated with impaired bone density and may be linked to higher risks of autoimmune and infectious diseases [109].

Adolescent are at high risk of iron deficiency and anaemia due to accelerated increase in requirements for iron, poor dietary intake of iron, high rate of infecton and worm infestation as well as the social norm of early marriage and adolescent pregnancy [110]. Iron deficiency is the leading contributor to adolescent disability-adjusted life years associated with micronutrient deficiencies [111]. In adolescent girls after onset of menarche, anaemia may impair immune system response which at times predisposes this vulerable group for increased morbidity and consequent poor health [104]. Suboptimal iron status and anaemia in adolescence can have significant effects on academic performance and school attendance due to symptoms of increased fatigue and susceptibility to infectious diseases [73]. Along with iron, folic acid is a very important B-complex vitamin that cannot be synthesized by the human body: therefore, it has to be obtained through the diet. Adolescence, which is the second period having the highest growth velocity after infancy, is the critical period to obtain adequate intake of folic acid. However its intake is generally low, and a dieficiency of this vitamin may lead to increased risk of cardiovascular diseases, major depression, schizophrenia, Alsheimer disease, and some carcinomas such as colorectal, uterine, cervial, lung and esophagus [112]. The most dramatic complication of folic acid deficiency is seen in infants whose mothers consumed inadequate amounts of folic acid early in pregnancy, resulting in neural tube defects (Krishnaswamy & Nair, 2001). In order to maintain the health and future of this vulnerable population, it is essential to take action to improve the iron and folic acid status of adolescents, especially among females who are at risk.

## Opportunities to Enhance the Health and Nutrition of Adolescent

### Adolescence as a Period of Significant Developmental Transition

Adolescence as a period of significant development transition: Adolescence is a period of significant development transition, a time of openness to new ideas, and a time of making personal choices rather than following family ptterns. It therefore presents a key opportunity for health and nutrition education interventions, with the potential to impact lifetime eating habits and health. Moreover, adolescents are often the first to adopt new technologies, particularly through the Internet which creates the opportinities for innovative delivery mechanisms [8]. The current generation of adolescents is termed the ‘do-it-yourself’ generation based on their increased sense of automomy and self-reliance due to a hgihtenend awareness of societal issues and the ability to immediately share their thoghts [113].

The expansion of transnational food and beverage coroarations into emerging markets is accelerationg the nutriton transition for adolescents by promoting the availability, affordability, and attractiveness of high-calorie ultra-processed foods. Given these increasingly transnational dimentions of the ultra-processed food industry, it is timely for WHO, the Food and Agricultural Organization of the UN, and their partners to revisit calls for global regulatory frameworks to assist governments in takeing action for the reduction of the consumption of products that are harmful to young people and the planet [114–116].

An unparalled opportunity to break the intergenerational cycles of malnutrition and respond to the urgent challenges of planetary chance is provided to adolescents. In addition to becoming customers, young people themselves are demanding roles. Food production already involves a large number of people. Yong people are increasingly becoming activits who draw attentioni to hpw the food industry and agriculture affect global ecosystems. Young people have the power to break the political and policy system around unhealthy food [117]. In addition to being the beneficiaries, adolescents will play a key role in enacting the revolutionary change requried to promote safe, substainable, and healthful food system(Patton et al., 2022).

### Building Better Food Environment

The present trends of change in the contemporary food environment are detrimental in their contribution to diet-related non-communicable diseases. The global food system urgently has to be changed in order to safeguard the palnet’s and people’s health and future [118]. Independent interaction with and exposure to the woder world occurs for the first time in adolescence, with this age group particularly susceptible to the structure, influences and offerings of the obesogenic food environment [119]. Adolescents are especially affected by their family, social, retail, and online food environments, each of which presents a different set of intervention opportunities.

Family- led intervention: Parents play a vital role in promoting children’s healthy eating and activity behaviors and preventing obesogenic environments for their children and adolescents. Good parenting behaviors may be correlated with improvement in adolescent’s healthy lifestyle behaviors and obesity status [120]. Family based behavioural interventions targeting deit, sedentary behaviours, physical activity and sleep qualtiy have been recommended as a first-line approact for the treatment of adolescent obesity and dietary improvement [98].

Peer-led intervention: Adolescents spend a substantial amount of time with friends, and eating is an important form of socialization and recreation. Because adolescents seek peer approval and social identity, it is assumed that peer infludnce and group conformity are important determinants in food acceptability and selection. Youth role models who can act as beacons of influence and positive peer modeling have been shown to improve dietary behavior, and peer-led interventions have been emphasized as feasible and acceptable among adolescents [121].

Environmental intervention: Enivronments such as school, followed by fast –food restaurants, vending machines, etc. have a large impact on adolescent eating, influencing access to and availability of foods and influencing perceived norms regarding eating behaviors. Thus environmental interventions such as the optimisation of schools and community food systems and regulation of the cost and marketing of food and drins may be effective in improving adolescent food choices [122]. The school food environment can have a large impact on adolescents’ food choices and dietary quality because adolescents consume a large proportion of their total daily energy at school.

Online food environment: Adolescents’ dietary preferences are significantly influenced by the layout and selection of the real and virtual retain food environments. Thorugh the promotion of fruits and vegetables, accessible nutrition labels, and ethical marketing practices food manufacturers and merchants have been working harder to promote healthy diet [123, 124]. However, several practices have been idenfitied as concerning, such as dispareate definations of ‘’healthy” products, a lack of progress on voluntary product reformulations, and a lack of adherence to guidelines for marketing products to young people [123]. Food retailers’ practices could be greatly improved, as some supermarkets target people in lower socio-economic areas with unhealthy food options, which is lieley contributing to obesity and other health problems in these areas [125]. Furthermore, compared to traditional media, online advertising of unhealthy foods is widespread and loosely controlled [126]. Due to their freqent usuage of social media and the internet, adolescents are frequently exposed to online food advertisements, which can have a big impact on their attitutdes and actions toward food [89]. As demonstrated by the decrease in consumption of sugar-seweetened beverages due to traffic light labeling, price increment and the promotion of healthier alternatives in retain settings, working wth food retailers to alter food environments has the potential to be very effective in encourageing more healthful dietary choices [127–129].

Society has an obligation to safeguard the health of adolescents and ensure the well-being of future generations. Creating supportive food environments that make healthy choices the easiest and most accessible options is crucial for protecting adolescent health. However, enhancing these food environments require actio nacross all areas, including the implementation of robust policies to encourage and oversee improvements in these sectors. This Table 1 summarizes research findings relevant to the opportunities for improving adolescent health and nutrition.

**Table 1:** Key research studies on opportunities to enhance adolescent nutrition and health

Study	Objective/Purpose	Key Findings
George C. Patton et al. (2016)	To explore adolescence as a critical developmental period for health and nutrition interventions.	Adolescence is an open period for shaping lifelong habits. Technology adoption creates opportunities for innovative interventions.
Singh & Dangmei (2016)	To analyze the autonomy and societal awareness of adolescents.	Adolescents, termed the “do-it-yourself” generation, are self-reliant and aware of societal issues, enabling impactful engagement.
Branca et al. (2020)	To evaluate the influence of global food systems on adolescent nutrition.	Transnational corporations accelerate the consumption of ultra-processed foods, increasing the need for global regulatory frameworks.
Horton (2019)	To highlight the role of adolescents in food activism and sustainable food systems.	Adolescents are demanding roles beyond consumers, advocating for sustainability and influencing food systems and policies.
Patton et al. (2022)	To emphasize adolescents’ potential in reshaping food systems for sustainability.	Adolescents can drive systemic changes, break cycles of malnutrition, and advocate for sustainable, healthful food systems.
Willett et al. (2019)	To evaluate global food system changes and their impact on health and sustainability.	Urgent changes in global food systems are needed to combat diet-related diseases and environmental harm.
Moore Heslin & McNulty (2023)	To analyze adolescents’ susceptibility to obesogenic food environments.	Adolescents are heavily influenced by food environments, highlighting the need for interventions across family, school, and online spaces.

## Policy Measures to Safeguard the Nutrition and Health of Adolescent

Early childhood stunting and wasting as well as child mortality have decreased as a result of nutritional expenditures made during the first 1000 days of life [130]. However, investments and interventions throughout later childhood and adolescence are required to end the malnutrition in all of its forms. Such investments will benefit later adult health and, in turn, the early growth and development of the next generation [5, 131]. To increase adolescent dietary choices and their effects, better national data system are required. These statistics, together with more solid information on enhancing adolescent nutriton in rapidily shifting food environment, these data will be essential to inform and strengthen policy making [116].

Adolescents’ nutritional shift is being accelerated by the entry of multinational food and beverage companies into emerging nations, which makes high-calorie, highly processed meals more accessible, affordable, and appealing. Local and national producers have imitated, making these items a staple in the diets of teenagers everywhere [132]. Unless any action is taken, the consumption of high-calorie, ultra-processed food is likely to increase, as well as the disruptions to food suply chains. Governments everywehre will need to adopt multiple and coordinated actions, including taxation, front of packet labelling, and regulation of marketing, to reduce the consumption of products that are harmful to young people and the planet. Beyond government, multiple stakeholders, including the food industry, will need to take action to reverse the increased consumption of unhealthy, ultra-processed foods and help end adolescent malnutrition [114].

Addressing adolescent nutrition presents an unparalleled opportunity to interrupt intergenerational cycle of malnutrition and repsond to the urgent challenges of planetary change. Young people themselves are demanding roles beyond being consumers. Many already participate in the production of food. A growing number of young people are activists who draw attention to how the agricultural and food industry affect the global ecosystems. Adolescents also have the potential to unlock the political and policy paralysis around unhealthy food systems [117]. Adolescents themselves should be at the centre of all interventions and should ideally be involved in each stage of the conception, research, planning, implementation, and evaluation of interventions to ensure they are understandable, applicable and effective for them [133]. This Table 2 summarizes research findings related to policies for protecting adolescent health and nutrition.

**Table 2:** Key research studies on policy measures to safeguard adolescent nutrition and health

Study	Objective/Purpose	Key Findings
Azzopardi et al. (2019)	To evaluate the impact of nutritional investments beyond early childhood.	Investments during adolescence, beyond the first 1,000 days, are critical for addressing malnutrition and improving intergenerational health outcomes.
Beal et al. (2021)	To study the influence of multinational corporations on adolescent diets.	High-calorie, ultra-processed foods are made more accessible and appealing by multinational corporations, driving unhealthy dietary transitions.
Branca et al. (2020)	To address the need for policies to limit the consumption of ultra-processed foods among adolescents.	Governments must adopt taxation, labeling, and marketing regulations to reduce harmful food consumption and protect adolescent health.
Patton et al. (2022)	To emphasize the importance of data-driven policymaking for adolescent nutrition.	Strong national data systems and coordinated actions are essential to improve adolescent dietary choices and reduce malnutrition.
Hargreaves et al. (2022)	To evaluate long-term benefits of addressing malnutrition during adolescence.	Addressing adolescent nutrition benefits not only individual health but also the growth and development of future generations.
Neveux et al. (2019)	To advocate for adolescent involvement in designing and implementing nutrition policies.	Adolescents’ active involvement in policy planning and evaluation ensures that interventions are relevant, effective, and impactful.
“Framework Convention for Obesity Control” (2011)	To propose a global framework for combating obesity through regulatory measures.	Calls for international regulations to address obesity, focusing on reducing the consumption of ultra-processed foods among adolescents.

## Conclusions

Investing in adolescent health and nutrition is one of the most impactful ways to safeguard the future. As global populations age and fertility rates decline, preventing youth health issues is crucial to preserving human potential. Adolescent health must extend beyond the purview of the health sector, requiring coordinated efforts from education, retail, government and infrastructure sectors to foster environments that support health and well-being. This review synthesizes the expanding body of research on adolescent nutrition and health, emphasizing the necessity of translating this evidence into actionable public health measures. Neglecting to address nutritional needs and dietary habits during adolescence can have serious consequences for lifelong health, well-being, and the resilience of future generations. Adolescents, as current and future leaders, need and deserve focused interventions that address their unique health requirements.

## Key References

- Daly AN, O’Sullivan EJ, Kearney JM. Considerations for health and food choice in adolescents. Proc Nutr Soc. 2022;81(1):75–86.
  - This review examines adolescent food choices, highlighting how body image concerns, taste preferences, cost, convenience, and social influences shape eating habits. It emphasizes that effective interventions must address teens’ nutritional needs while considering these factors to promote both healthy eating and positive food relationships during this formative developmental period.
- The Lancet Child Adolescent Health. The hidden crisis of adolescent nutrition. Lancet Child Adolesc Health. 2022;6(1):1.
  - This editorial examines adolescent malnutrition as a neglected global crisis, emphasizing adolescence as a critical nutritional period. It identifies unhealthy food environments and targeted marketing as key challenges while calling for better data, regulation, and youth engagement to address this urgent public health issue.
- Patton GC, et al. Nourishing our future: the Lancet Series on adolescent nutrition. Lancet. 2022;399(10320):123–125.
  - This passage highlights adolescent nutrition as a neglected priority despite being a critical growth period second only to early childhood. It identifies key policy gaps: adolescents are missing from major global nutrition initiatives and targets. Meanwhile, adolescent health faces a dual burden - overweight/obesity has more than doubled (affecting nearly one in five globally) while anemia has increased by 20% (affecting almost one in four), yet donor funding remains inadequate.

## Data Availability

No datasets were generated or analysed during the current study.
